# Seroprevalence of *Neospora caninum* Infection in Shelter Dogs from Hanoi, Vietnam

**DOI:** 10.3390/ani16081205

**Published:** 2026-04-15

**Authors:** Nhung Pho Nguyen Nguyen, Hanh Thi Ha, Bach Xuan Pham, Eukote Suwan, Ketsarin Kamyingkird, Chanya Kengradomkij, Charoonluk Jirapattharasate, Tawin Inpankaew

**Affiliations:** 1 Department of Parasitology, Faculty of Veterinary Medicine, Kasetsart University, Bangkok 10900, Thailand; nhungphonguyen.n@ku.th (N.P.N.N.); hanhthi.h@ku.th (H.T.H.); bachxuan.p@ku.th (B.X.P.); ketsarin.ka@ku.th (K.K.); fvetcyk@ku.ac.th (C.K.); 2 Department of Veterinary Technology, Faculty of Veterinary Technology, Kasetsart University, Bangkok 10900, Thailand; eukote.su@ku.th; 3 Department of Pre-Clinic and Applied Animal Science, Faculty of Veterinary Science, Mahidol University, 999 Phuthamonthon Sai 4 Rd, Salaya, Nakhon Pathom 73170, Thailand

**Keywords:** *Neospora caninum*, recombinant protein, dense granules, serology, dogs, Vietnam

## Abstract

Neosporosis, caused by *N. caninum*, is an emerging protozoan disease responsible for significant economic losses in the global dairy and meat industries, primarily due to abortion in cattle. Dogs serve as both definitive and intermediate hosts and play a key role in the parasite transmission cycle. Currently, effective control strategies remain limited, partly due to insufficient information on infection status. In Vietnam, data on *N. caninum* infection are scarce and mainly limited to cattle and buffalo. In this study, an indirect enzyme-linked immunosorbent assay (iELISA) based on recombinant NcGRA4 protein was applied and evaluated for the detection of anti-*N. caninum* antibodies in dogs. A total of 142 shelter dogs from Hanoi, northern Vietnam, were tested to determine seroprevalence. The NcGRA4-based iELISA detected an overall seroprevalence of 28.87% (41/142), whereas the indirect fluorescent antibody test (iFAT) showed a lower prevalence of 14.08% (20/142), indicating substantial exposure to *N. caninum* among shelter dogs in this region. Using iFAT as the reference method, the NcGRA4-based iELISA demonstrated a sensitivity of 90.00%, a specificity of 81.15%, and an overall accuracy of 82.39%. These findings indicate that the NcGRA4-based iELISA is a suitable screening tool for seroepidemiological surveillance of *N. caninum* infections in dogs. Univariable and multivariable logistic regression analyses showed no significant associations between *N. caninum* seropositivity and the investigated variables, including age, sex, breed, and housing conditions. This study also provides the first serological evidence of canine exposure to *N. caninum* in Vietnam.

## Simple Summary

Neosporosis is a parasitic disease that can cause abortion in cattle and lead to major economic losses in livestock production. Dogs play an important role in spreading this parasite in the environment. However, information about infection in dogs in Vietnam is very limited. In this study, we examined blood samples from 142 shelter dogs in Hanoi to determine how commonly they had been exposed to the parasite. We used a recombinant protein NcGRA4-based indirect enzyme-linked immunosorbent assay (iELISA) to detect antibodies, which indicate previous exposure. Nearly one-third of the tested dogs showed evidence of exposure, suggesting that the parasite is circulating among dogs in this area. We also compared this method with the indirect fluorescent antibody test (iFAT) and found that it may be useful as a large-scale screening tool for *Neospora caninum* infection in dogs. Our findings provide new information about the presence of this parasite in dogs in northern Vietnam and may help improve disease monitoring and control strategies to reduce the risk of infection in livestock.

## 1. Introduction

Neosporosis is an intracellular protozoan disease caused by *Neospora caninum*, an Apicomplexan parasite characterized by its broad host range and global distribution [1]. Canids serve as both definitive and intermediate hosts, playing a central role in parasite transmission through oocyst shedding and vertical transmission [2]. A wide variety of domestic and wild animals act as intermediate hosts, among which cattle, goats, and sheep are of particular importance [3,4]. In these hosts, neosporosis is widely recognized as a major infectious cause of abortion and is associated with considerable economic losses to the livestock sector due to reproductive failure, reduced milk production, and expenses related to disease control [2,4,5].

Several diagnostic methods are available for the detection of *N. caninum* infection. In cases of bovine abortion, histopathology, immunohistochemistry (IHC), and polymerase chain reaction (PCR) performed on fetal tissues are regarded as confirmatory diagnostic techniques [6,7]. Nevertheless, the high costs, technical demands, and need for specialized laboratory facilities restrict their applicability in large-scale epidemiological surveillance [8]. Consequently, serological assays that detect anti-*N. caninum* antibodies are widely used for epidemiological investigations and routine diagnosis [9]. Serological techniques, including the indirect fluorescent antibody test (iFAT), enzyme-linked immunosorbent assay (ELISA), and Western blot, are considered effective for the detection of *N. caninum* antibodies in both field and experimental animals when potent and specific antigens are used [10]. Although *Neospora* lysate antigen (NLA) has been widely used as a reference antigen, recombinant antigens offer improved standardization, specificity, and reproducibility [9].

The adhesion, invasion, and intracellular survival of *N. caninum* involve a coordinated process mediated by the apical complex and associated secretory organelles, including surface proteins, rhoptries, micronemes, and dense granules [11,12]. Dense granule proteins (GRAs) are abundantly secreted after host cell invasion and are integral components of the parasitophorous vacuole and cyst wall, making them suitable targets for immunological studies, vaccine development, and serodiagnosis [11,13]. Several GRAs, including NcGRA2 [14], NcGRA6 [15,16], NcGRA7 [8,15,16], NcGRA9 [17], and NcGRA14 [18], have been identified, with NcGRA6, NcGRA7, and NcGRA14 demonstrating promising diagnostic potential in bovine neosporosis. Recently, NcGRA4 has received considerable attention due to its strong immunogenicity, its role in intracellular parasite survival, and its favorable diagnostic performance, including encouraging sensitivity and specificity reported in a small ruminant model [11,19].

Globally, serological surveys have shown highly variable exposure rates of *N. caninum* in canine populations [20]. In Vietnam, available data on *N. caninum* infection remain limited and are largely restricted to studies in livestock. Early serological surveys reported a seroprevalence of 5.5% in cattle and 1.5% in water buffaloes in southern Vietnam [21], while a subsequent study reported a substantially higher prevalence of 41% in dairy cattle from the same region [22]. In northern Vietnam, *N. caninum* antibodies were detected in 30% of lactating dairy cattle in Hanoi [23]. To date, however, no seroepidemiological study using standardized serological methods has been conducted to investigate *N. caninum* infection in the canine population in Vietnam. In addition, identifying potential risk factors associated with *N. caninum* infection is important for understanding its epidemiology and transmission.

In Vietnam, including the north, livestock production is commonly integrated with smallholder farming systems, where cattle, buffaloes, pigs, and dogs often coexist in close proximity, potentially facilitating the transmission of infectious agents between species [24]. Hanoi, the capital of Vietnam, is experiencing rapid urbanization, characterized by high population density and large numbers of domestic animals [25]. The city also hosts numerous dog slaughterhouses that supply meat to nearby areas [25]. Since dog meat consumption remains legal in Vietnam and is considerably more common in the north than in the south [26], demand for dog meat in Hanoi is particularly high. Consequently, widespread dog theft and illegal trading have become increasingly prevalent. Many dogs rescued from these illegal activities are placed in shelters, leading to overcrowding and strain on facilities with limited financial and veterinary resources. These conditions pose challenges for animal welfare and disease prevention, making shelter dogs a population of epidemiological concern.

Therefore, the present study aimed to determine the seroprevalence of *N. caninum* infection among shelter dogs in northern Vietnam using a recombinant NcGRA4-based indirect enzyme-linked immunosorbent assay (iELISA) and the indirect fluorescent antibody test (iFAT), and to evaluate potential risk factors associated with seropositivity. This study represents the first seroepidemiological investigation of canine neosporosis in Vietnam and provides baseline data for future epidemiological studies and diagnostic development.

## 2. Materials and Methods

### 2.1. Ethical Statement

All procedures involving animals were conducted in accordance with institutional guidelines for animal care and use. The study protocol was reviewed and approved by the Kasetsart University’s Institutional Animal Care and Use Committee (Approval number ACKU67-VET-099).

### 2.2. Study Period and Location

The sampling was conducted in February 2025 in Hanoi, the capital city of Vietnam, located in the northern part of the country. Hanoi lies at the center of the Red River Delta and is characterized by lowland plains in the central areas, with hilly and mountainous terrain in the northern and western parts of the city.

**Figure 1:**
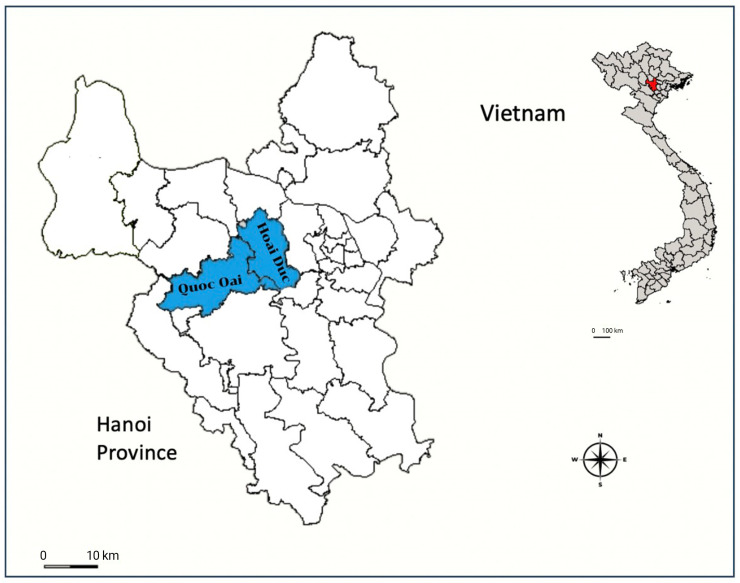
A map showing sampling locations within Hanoi City, Vietnam. The markers’ color indicates the specific areas under investigation in this study.

The study sites were selected to represent different dog populations and were located in suburban districts outside of the city center, where small-scale livestock farming, particularly cattle production, is common. A total of 142 shelter dogs were collected from two major animal rescue centers, including 68 samples from Hoai Duc District and 74 samples from Quoc Oai District, Hanoi, Vietnam (Figure 1).

### 2.3. Sample Collection

The sample size was calculated using a regional prevalence estimate of 7.7% for *N. caninum* infection in dogs in Southeast Asia [20], with a 95% confidence level (*Z*) and a margin of error of 5% (*E*), using the single-proportion formula:

$$n=\frac{Z^{2}\;\times \;p\;\times \;(1-p)}{E^{2}}$$

Although the minimal sample size calculated for this study was 110 animals, we increased the number of samples collected to improve the degree of accuracy and to account for some potential sample losses. Consequently, a total of 142 blood samples were collected.

Blood samples (approximately 1–2 mL) were obtained by puncture of the cephalic vein using sterile 23-gauge needles and collected into vacuum tubes without anticoagulant. Serum was separated after clotting and centrifugation, then stored at −20 °C until serological testing.

Sampling was conducted using a convenience approach, whereby all available dogs at the time of sampling were included, while ensuring equal representation from each housing unit. Most dogs were clinically healthy at the time of sampling, although a small number were mildly emaciated or had recently recovered from illness.

Basic information, including age, sex, breed, and housing conditions, was recorded for each dog. Dogs were housed within the shelter facility and had no direct contact with livestock during the study period. However, information regarding prior exposure to livestock before admission to the shelter was not available.

### 2.4. Production of Recombinant NcGRA4 Protein

The recombinant NcGRA4 protein was expressed and purified following previously described protocols [19]. Briefly, a truncated fragment of the NcGRA4 gene corresponding to amino acids 24–263 was cloned into the pET-21d expression vector containing an N-terminal FLAG tag (DYKDDDDK) using the NcoI and XhoI restriction sites.

The recombinant NcGRA4 plasmid (10 ng/μL) was transformed into *Escherichia coli* BL21 (DE3) cells for protein expression. Transformed bacterial cells were cultured in 2XYT broth supplemented with 100 μg/mL ampicillin and incubated overnight at 37 °C with shaking at 200 rpm. The cultures were then transferred into fresh 2XYT broth and grown at 37 °C with shaking until the optical density at 600 nm (OD_600_) reached approximately 0.6.

Expression of the NcGRA4 protein was induced by adding isopropyl β-D-thiogalactopyranoside (IPTG) to a final concentration of 1 mM, followed by incubation at 25 °C overnight with shaking at 200 rpm. The induced bacterial cells were harvested by centrifugation, and the cell pellet was lysed using BugBuster^TM^ Protein Extraction Reagent (Novagen, Darmstadt, Germany).

The recombinant NcGRA4 protein was purified using anti-DYKDDDDK agarose beads (Smart Lifescience, Hong Kong, China). Purified protein fractions were pooled and dialyzed using SnakeSkin Dialysis Tubing (10 kDa cut-off) against phosphate-buffered saline (PBS, pH 7.4) and stored at −20 °C until use. The purity and integrity of the recombinant protein were confirmed by 12% sodium dodecyl sulfate–polyacrylamide gel electrophoresis (SDS-PAGE) and Western blot analysis (see Supplementary Figure S1).

### 2.5. NcGRA4-Based Indirect ELISA

The optimal conditions for the NcGRA4-based indirect enzyme-linked immunosorbent assay (iELISA) were determined by evaluating different antigen concentrations and serum dilutions. All canine serum samples were tested in duplicate.

Briefly, ELISA strip microplates (BKMAM Biotech, Changde, Hunan, China) were coated with 100 μL per well of recombinant NcGRA4 protein at a final concentration of 1 or 2 μg/mL diluted in 1× ELISA coating buffer (Bio-Rad, Hercules, CA, USA) and incubated overnight at 4 °C. Plates were washed five times with phosphate-buffered saline containing 0.1% Tween-20 (PBS-T). Subsequently, 100 µL of canine serum samples diluted 1:100 or 1:200 in 1× BlockPro^TM^ Blocking buffer (Energenesis Biomedical, Taipei, Taiwan) were added to each well and incubated for 1 h at room temperature. After washing five times with PBS-T, 100 µL of horseradish peroxidase goat anti-dog IgG (H + L) antibodies (Bio-Rad, Hercules, CA, USA), diluted 1:8000 in BlockPro^TM^ buffer, were added to each well and incubated for 1 h at room temperature.

After another five washes with PBS-T, 50 µL of 3,3′,5,5′-tetramethylbenzidine (TMB) substrate (KPL, SeraCare Life Sciences, Milford, MA, USA) was added to each well for color development. The reaction was stopped after 15 min by adding 50 µL of 0.1 M HCl, following the protocol described by Udonsom et al. (2023) [19]. Optical density (OD) values were measured at 450 nm using a spectrophotometer (Multiskan RC, Thermo Labsystems, Vantaa, Findland).

### 2.6. Indirect Fluorescent Antibody (iFAT)

All serum samples were examined using the indirect fluorescent antibody test (iFAT) to confirm infection status and to compare the results with those obtained by iELISA. iFAT antigen slides were prepared using tachyzoites of the *N. caninum* Nc-1 strain, as described previously [27].

Canine serum samples were initially diluted 1:50 [28] in phosphate-buffered saline (PBS) containing 5% bovine serum albumin (BSA) and incubated on *Neospora* antigen-coated slides at 37 °C for 1 h. Slides were then incubated with fluorescein isothiocyanate (FITC)-conjugated goat anti-dog IgG (Catalog No. 035-10, VMRD, Pullman, Washington, USA) at 37 °C for an additional 1 h.

**Figure 2:**
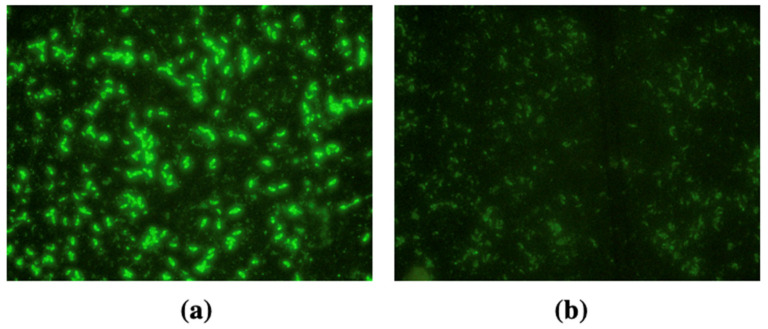
Positive dog sample in the iFAT for anti-*N. caninum* IgG, demonstrating extensive peripheral fluorescence of *N. caninum* tachyzoites (**a**), and a negative dog sample for anti-*N. caninum* IgG displayed an absence of fluorescein signal (**b**) visualized under a ×40 objective.

Commercial *N. caninum* positive and negative control sera (VMRD, Pullman, Washington, USA) were included in each test. Slides were examined under a fluorescence microscope, and samples showing a bright, linear peripheral fluorescence of the tachyzoites were considered positive, whereas the absence of fluorescence was indicated as negative (Figure 2). Samples positive at the 1:50 cut-off dilution were further subjected to serial two-fold dilutions to determine endpoint antibody titers.

### 2.7. Statistical Analysis

Statistical analyses were performed using SPSS Statistics software (version 27.0; IBM Corp., Armonk, NY, USA). Receiver Operating Characteristic (ROC) curve analysis was used to determine the optimal cut-off value of the NcGRA4-based iELISA using iFAT as the reference standard. Diagnostic performance was evaluated by calculating the area under the curve (AUC), sensitivity (Se), specificity (Sp), positive predictive value (PPV), negative predictive value (NPV), and kappa coefficient (κ) based on 2 × 2 contingency tables. Associations between potential risk factors and *N. caninum* seropositivity were assessed using the univariable and multivariable logistic regression analysis. A *p* < 0.05 was considered statistically significant.

## 3. Results

### 3.1. Optimization of the NcGRA4-Based iELISA

The optimal concentration of the purified NcGRA4 antigen was determined using concentrations of 1 and 2 μg/mL along with serum dilutions of 1:100 and 1:200, as shown in Table 1. As determined by the checkerboard assay, the pure NcGRA4 protein concentration of 1 μg/mL and the sample dilution of 1:200 were employed. These conditions provided the most acceptable positive-to-negative (P/N) ratio while using the lowest antigen concentration, thereby improving assay efficiency and reducing antigen consumption. Although relatively high background reactivity was observed in negative sera, these conditions were considered an optimal compromise between signal intensity and background noise. All optimization experiments were performed in duplicate, and consistent trends were observed across replicates.

### 3.2. Diagnostic Performance and Validation of the NcGRA4-Based iELISA

The diagnostic performance of the NcGRA4-based iELISA was evaluated using 142 canine serum samples with known *N. caninum* antibody status as determined by IFAT. In comparison with iFAT, 18 samples were positive by both methods, while 2 samples were positive by iFAT but negative by iELISA. In contrast, 23 samples were positive only by iELISA. Among the 122 iFAT-negative samples, 99 samples were correctly classified as negative by iELISA (Table 2).

**Figure 3:**
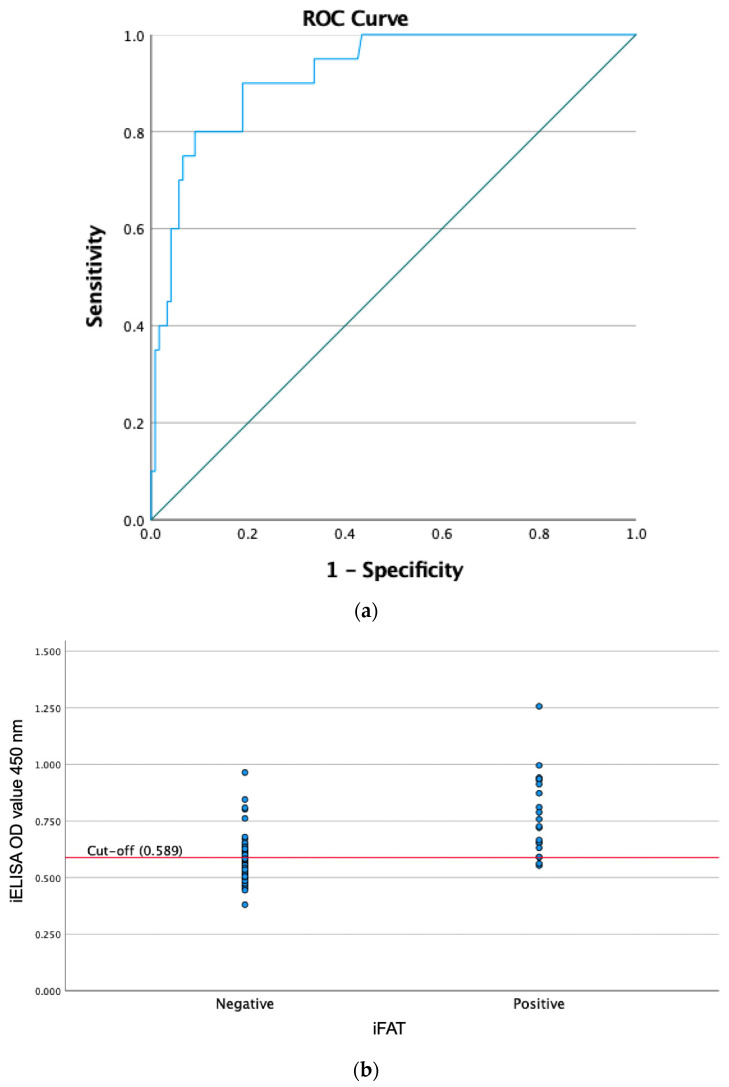
Receiver Operating Characteristics (ROC) analysis of the NcGRA4-based iELISA. (**a**) ROC curve (blue line) with the diagonal line indicating the reference line. (**b**) Distribution of iELISA optical density (OD) values according to iFAT results with the red horizontal line indicates the cut-off value.

Receiver operating characteristic (ROC) curve analysis (Figure 3a) yielded an area under the curve (AUC) of 0.92 (95% CI: 0.86–0.97). The optimal cut-off value (OD = 0.589) was determined by maximizing the Youden index (sensitivity + specificity − 1), providing the best balance between sensitivity and specificity. At this cut-off, the assay showed a sensitivity of 90.00% and a specificity of 81.15%. Using iFAT as the reference test, the iELISA demonstrated a positive predictive value (PPV) of 43.90%, a negative predictive value (NPV) of 98.02%, and an overall diagnostic accuracy of 82.39% (95% CI: 75.12–88.27%). The agreement between iELISA and iFAT was moderate, with a Cohen’s kappa value of 0.49 (Table 2). The distribution of iELISA OD values according to iFAT results was illustrated in Figure 3b, where partial overlap between iFAT-positive and iFAT-negative samples can be observed around the selected cut-off value.

### 3.3. Seroprevalence of N. caninum Infection in Dogs

Overall, the seroprevalence of *N. caninum* infection determined by iFAT was 14.08% (95% CI: 8.60–21.75%), whereas the NcGRA4-based iELISA detected a higher seroprevalence of 28.87% (95% CI: 20.72–39.17%).

**Figure 4:**
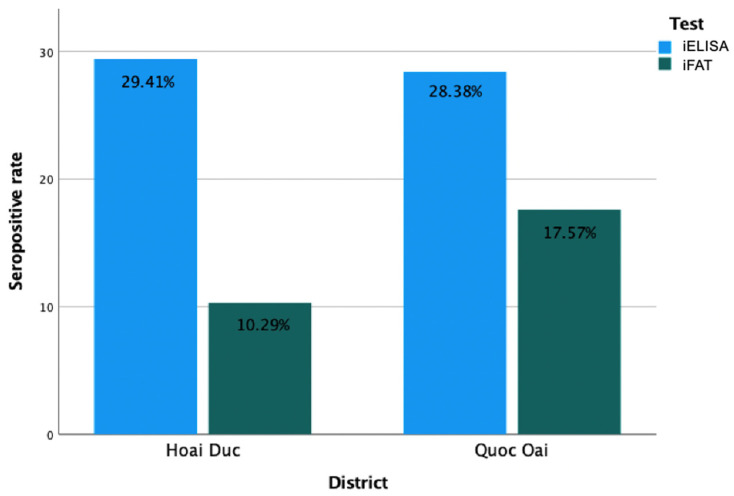
Seropositive rates of *N. caninum* in shelter dogs detected by iFAT and iELISA-NcGRA4 from investigated districts.

At the district level, iFAT-based seroprevalence was 17.57% (95% CI: 9.35–30.04%) in Quoc Oai District and 10.29% (95% CI: 4.14–21.21%) in Hoai Duc District. In contrast, iELISA-NcGRA4 identified a greater number of seropositive sera in both districts, with comparable seroprevalence rates of 28.38% (95% CI: 17.57–43.38%) and 29.41% (95% CI: 17.97–45.42%) in Quoc Oai and Hoai Duc districts, respectively. In general, iELISA-NcGRA4 detected a higher seroprevalence of *N. caninum* than iFAT in both districts (Figure 4).

### 3.4. iFAT Antibody Titer Distribution in Seropositive Dogs

Among the 20 iFAT-seropositive dogs, antibody titers against *N. caninum* ranged from 1:100 to 1:1600. Most positive samples showed moderate titers, predominantly 1:200 (11/20), followed by 1:400 (4/20). In Hoai Duc District (*n* = 7), titers were limited to 1:200 and 1:400, whereas a broader distribution (1:100–1:1600) was observed in Quoc Oai District (*n* = 13). High titers (≥1:800) were observed in only two dogs. Seropositivity did not differ significantly between districts (*p* = 0.21).

### 3.5. Risk Factors Associated with N. caninum Seropositive Dogs

Univariable analysis showed that none of the evaluated factors, including sex, age, breed, and housing conditions, were significantly associated with *N. caninum* seropositivity (*p* > 0.05) (Table 3).

## 4. Discussion

Diagnosis of *N. caninum* infection in dogs primarily relies on serological assays that have been used as important tools for both clinical and seroepidemiological investigations [28]. Nevertheless, enhancing diagnostic accuracy remains essential to optimize diagnostic sensitivity and specificity, particularly due to the potential for serological cross-reactivity with closely related protozoan parasites [29]. In this study, we evaluated the diagnostic performance of a recombinant NcGRA4-based iELISA in detecting *N. caninum*-specific IgG antibodies in dogs from northern Vietnam, using iFAT as the reference method. A cut-off titer of 1:50, commonly recommended for canine sera [28], was adopted to define iFAT seropositivity. The NcGRA4-based iELISA demonstrated good overall discriminatory ability, with an area under the ROC curve of approximately 0.92. At the selected cut-off value, the assay showed high sensitivity (90.00%), suggesting that this assay may serve as a useful screening tool in canine populations for seroepidemiological surveys. However, despite this favorable ROC profile, the assay showed only moderate specificity (81.15%), and moderate agreement with iFAT (κ = 0.49), indicating that the practical diagnostic performance of the NcGRA4-based iELISA should be interpreted with caution and that it may not be suitable as a standalone confirmatory test. Examination of the OD distribution revealed partial overlap between iFAT-positive and iFAT-negative sera around the selected cut-off value. Such overlap may lead to misclassification of some samples and likely contributed to the occurrence of ELISA-positive/iFAT-negative results. In addition, relatively elevated OD values observed in some negative sera during assay optimization suggest the presence of background reactivity, which may partially contribute to reduced specificity and the occurrence of false-positive ELISA results. Further optimization of assay conditions, including antigen concentration, blocking reagents, and serum dilution, may help reduce nonspecific reactivity and improve the specificity of the NcGRA4-based iELISA in future applications.

A notable finding of this study was the relatively high number of ELISA-positive but iFAT-negative samples. This discrepancy may partly reflect the higher analytical sensitivity of recombinant antigen-based ELISAs in detecting low-level responses, including those present during early-stage infections [16,30], which may remain below the detection threshold of iFAT. Nevertheless, the possibility of false-positive ELISA results cannot be excluded. Differences in antigen composition between whole-parasite iFAT and recombinant antigen-based iELISA, nonspecific antibody binding, or potential cross-reactivity with antibodies against other antigenically related Apicomplexan parasites (e.g., *Toxoplasma gondii* or *Sarcocystis* spp.) may contribute to such discordant results [29,31]. Previous studies evaluating recombinant NcGRA4 in goats reported no cross-reactivity with *T. gondii*-positive goat sera, supporting the antigenic specificity of NcGRA4 [19]. However, cross-reactivity was not directly assessed in the present dog study, and the potential influence of antibodies against other protozoan parasites cannot be completely excluded. Without molecular confirmation or longitudinal follow-up, it is not possible to determine whether these ELISA-positive/iFAT-negative samples represent true infections or false-positive reactions.

The relatively low positive predictive value (PPV, 43.90%) observed in this study should be interpreted in the context of both disease prevalence and test performance. PPV is strongly influenced by the underlying prevalence of infection, and lower PPVs are expected in low-prevalence settings even when test sensitivity is high [32]. Importantly, PPV is also strongly affected by test specificity. In the present study, the moderate specificity of the NcGRA4-based iELISA may have contributed to the reduced PPV, as lower specificity increases the likelihood of false-positive results. Consequently, a proportion of ELISA-positive samples, particularly those that were negative by iFAT, may represent nonspecific reactions rather than true infections, which could partly lead to an overestimation of seroprevalence. In contrast, the excellent negative predictive value (NPV, 98.02%) indicates that a negative ELISA result reliably excludes infection as defined by iFAT. These findings further support the potential utility of the NcGRA4-based iELISA as a screening tool, although positive results should be interpreted with caution.

The overall seroprevalence of *N. caninum* infection detected by NcGRA4-based iELISA (28.87%) was notably higher than that determined by iFAT (14.08%), a pattern consistent with previous studies comparing ELISA and iFAT-based assays [20]. The higher detection rate observed with iELISA may reflect the increased analytical sensitivity of ELISA-based methods, particularly when recombinant antigens are employed. However, it may also be influenced by reduced specificity and the presence of false-positive results, potentially leading to an overestimation of seroprevalence. When compared with regional and international data, the seroprevalence observed in the present study exceeded the overall estimate reported for Southeast Asia (7.70%) [20], as well as the rates documented in Indonesia (3.4%) [33], Thailand (3.8–15.7%) [34,35,36], China (15–20%) [37,38], Korea (3.6%) [39], Ecuador (6.8%) [40], Brazil (12.4%) [41], and Poland (1.0–21.7%) [42,43]. In contrast, substantially higher prevalence levels have been described in Jordan (34.3–47.9%) [44] and in Australia (29.8–32.8%) [45], while the estimate reported in Iraq (26.7%) [46] was comparable to our findings. The variability in seroprevalence across countries likely reflects differences in climatic and ecological conditions, livestock density, dog management practices, and the extent of environmental contamination with oocysts. Additionally, discrepancies in study design, characteristics of sampled dog populations (e.g., stray versus owned or rural versus urban dogs), and diagnostic methodologies, antigen selection, and cut-off thresholds may substantially influence reported prevalence estimates [20,47,48]. To the best of our knowledge, this study represents the first serological evidence of canine exposure to *N. caninum* in Vietnam, thereby contributing important epidemiological data to the regional understanding of canine neosporosis.

Analysis of iFAT antibody titers revealed that most seropositive dogs had low to moderate titers, predominantly between 1:200 and 1:400. Such titers are commonly associated with past exposure or possibly chronic infection, which may be consistent with the largely subclinical nature of *N. caninum* infection in adult dogs [28]. However, it should be emphasized that antibody titers alone cannot reliably distinguish between infection stages. The broader range of titers observed in Quoc Oai District compared to Hoai Duc District may reflect differences in exposure intensity or timing; however, the absence of a statistically significant difference in overall seropositivity between districts (*p* = 0.21) suggests that infection pressure is likely comparable across the two areas. These findings support the hypothesis that *N. caninum* exposure among shelter dogs is relatively homogeneous, possibly due to similar background histories prior to shelter admission, shared environmental conditions, or management practices. Nevertheless, longitudinal studies incorporating clinical and molecular data would be required to better understand infection dynamics and the clinical relevance of varying antibody titers.

In the present study, none of the evaluated variables, including age, sex, breed, and housing conditions, were significantly associated with *N. caninum* seropositivity. Similar findings have been reported in some previous studies, where no significant associations were observed between demographic factors and canine neosporosis [36,49]. In contrast, other studies have identified certain host- or environment-related factors as potential risk determinants [37,45,50], suggesting that the epidemiology of *N. caninum* infection in dogs may vary depending on local conditions and study design. The lack of significant associations in the present study may be explained by several factors. First, the relatively limited sample size may have reduced the statistical power to detect weak or moderate associations. Second, the study population consisted exclusively of shelter dogs, which are likely to share similar management conditions, feeding practices, and environmental exposures, resulting in a relatively homogeneous population. Such homogeneity may obscure potential differences between groups. In addition, the absence of detailed information on the dogs’ history prior to shelter admission may have limited the ability to identify relevant exposure-related risk factors.

Several limitations of this study should be acknowledged. First, the shelter-based sampling strategy may introduce selection bias and may not fully represent the broader canine population in northern Vietnam, thereby limiting the generalizability of the findings. Second, the lack of molecular confirmation, such as PCR-based detection, limits the ability to verify true infection status and to distinguish between active and past infections, particularly for discordant ELISA-positive/iFAT-negative samples. Third, relatively high background reactivity observed during assay optimization may have contributed to reduced specificity and the occurrence of false-positive ELISA results. In addition, potential cross-reactivity with antibodies against other apicomplexan parasites cannot be excluded and may have influenced the diagnostic performance of the NcGRA4-based iELISA. Fourth, the analysis of potential risk factors was limited by the availability and completeness of collected data. Finally, the cross-sectional design precludes assessment of temporal antibody dynamics and infection progression. Future studies incorporating molecular diagnostics, improved assay optimization, longitudinal sampling, and more comprehensive data on potential risk factor are needed to better understand the epidemiology and transmission dynamics of canine neosporosis in Vietnam.

## 5. Conclusions

In conclusion, the recombinant NcGRA4-based iELISA demonstrated good diagnostic performance and moderate agreement with iFAT, supporting its potential utility as a screening tool for epidemiological surveillance and large-scale detection of *N. caninum* infection in dogs. Although no significant risk factors were identified in this study, the findings provide important baseline epidemiological data. This study provides the first serological evidence of canine exposure to *N. caninum* in Vietnam and contributes valuable baseline data for future epidemiological studies and control strategies.

## Figures and Tables

**Table 1:** Checkerboard assay to verify the optimal concentration of purified NcGRA4 antigen and dilution of dog sera for iELISA.

	Sample Dilution	Concentration of Purified NcGRA4 Antigen	
		1 (μg/mL)	2 (μg/mL)
Positive	1:100	1.532	1.528
Negative		0.727	0.715
Positive	1:200	1.334	1.333
Negative		0.610	0.632

**Table 2:** Comparison of iFAT and NcGRA4-based iELISA for detection of anti-*Neospora caninum* IgG antibodies in dogs.

iFAT	iELISA-NcGRA4			Sensitivity	Specificity	PPV	NPV	Kappa Value
	Positive	Negative	Total					
Positive	18	2	20	90.00% (68.30–98.77%)	81.15% (73.07–87.66%)	43.90% (34.50–53.77%)	98.02% (92.98–99.46%)	0.49
Negative	23	99	122					
Total	41	101	142					

**Table 3:** Univariable logistic regression analysis of potential risk factors associated with *N. caninum* seropositivity.

Parameter	Category	iFAT+/Total (%)	OR (95% CI)	*p*-Value
Sex	Female	11/73 (15.07%)	(Ref)	
	Male	9/69 (13.04%)	1.23 (0.46–3.3)	0.68
Age	>5 years	4/31 (12.90%)	(Ref)	
	<12 months	4/33 (12.12%)	0.66 (0.14–3.25)	0.61
	1–5 years	12/78 (15.38%)	0.97 (0.26–3.65)	0.96
Breed	Exotic	5/40 (12.50%)	(Ref)	
	Domestic	12/85 (14.12%)	0.92 (0.27–3.11)	0.89
	Mixed	3/17 (17.65%)	1.19 (0.23–6.00)	0.84
Housing	Multi-cage	19/116 (16.38%)	(Ref)	
	Single-cage	1/26 (3.84%)	0.19 (0.02–1.52)	0.11
Total		20/142 (14.08%)		

## Data Availability

The original contributions presented in this study are included in the article. Further inquiries can be directed to the corresponding author.

## Supplementary Materials

The following supporting information can be downloaded at: https://www.mdpi.com/article/10.3390/ani16081205/s1, Figure S1: Expression and purification of recombinant NcGRA4 protein. Lane M: protein molecular weight marker; Lane 1: the eluted recombinant NcGRA4; Lane 2: the purified recombinant NcGRA4. The expected 32-kDa NcGRA4 band is indicated by a black arrow.

## Abbreviations

The following abbreviations are used in this manuscript:

CI	Confidence interval
GRAs	Dense granule protein
iELISA	Indirect Enzyme-linked immunosorbent assay
iFAT	Indirect fluorescent antibody test
