# Supplementary material for: Seroprevalence of Neospora caninum Infection in Shelter Dogs from Hanoi, Vietnam
Source: Animals (Basel). 2026 Apr 15;16(8):1205. doi: 10.3390/ani16081205 (PMC13114095; doi:10.3390/ani16081205)
Supplement: Supplementary file 1 [file animals-16-01205-s001.zip › animals-4214788-supplementary.pdf]

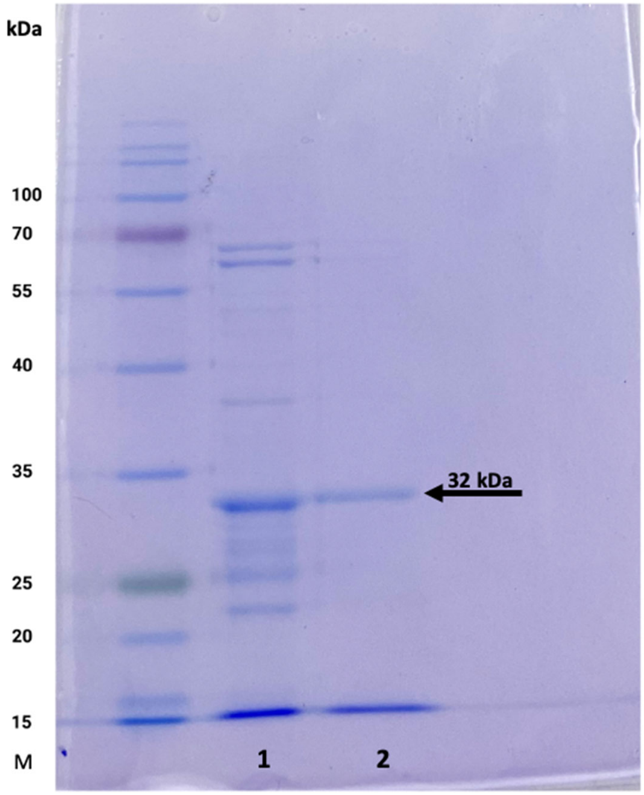

Figure S1. Expression and purification of recombinant NcGRA4 protein. Lane M: protein molecular weight marker; Lane 1: the eluted recombinant NcGRA4; Lane 2: the purified recombinant NcGRA4. The expected 32-kDa NcGRA4 band is indicated by a black arrow.
